# Miniature Transposable Sequences Are Frequently Mobilized in the Bacterial Plant Pathogen *Pseudomonas syringae* pv. phaseolicola

**DOI:** 10.1371/journal.pone.0025773

**Published:** 2011-10-10

**Authors:** Leire Bardaji, Maite Añorga, Robert W. Jackson, Alejandro Martínez-Bilbao, Natalia Yanguas-Casás, Jesús Murillo

**Affiliations:** 1 Laboratorio de Patología Vegetal, Departamento de Producción Agraria, Escuela Técnica Superior de Ingenieros Agrónomos, Universidad Pública de Navarra, Pamplona, Spain; 2 School of Biological Sciences, University of Reading, Reading, United Kingdom; University of Poitiers, France

## Abstract

Mobile genetic elements are widespread in *Pseudomonas syringae*, and often associate with virulence genes. Genome reannotation of the model bean pathogen *P. syringae* pv. phaseolicola 1448A identified seventeen types of insertion sequences and two miniature inverted-repeat transposable elements (MITEs) with a biased distribution, representing 2.8% of the chromosome, 25.8% of the 132-kb virulence plasmid and 2.7% of the 52-kb plasmid. Employing an entrapment vector containing *sacB*, we estimated that transposition frequency oscillated between 2.6×10^−5^ and 1.1×10^−6^, depending on the clone, although it was stable for each clone after consecutive transfers in culture media. Transposition frequency was similar for bacteria grown in rich or minimal media, and from cells recovered from compatible and incompatible plant hosts, indicating that growth conditions do not influence transposition in strain 1448A. Most of the entrapped insertions contained a full-length IS*801* element, with the remaining insertions corresponding to sequences smaller than any transposable element identified in strain 1448A, and collectively identified as miniature sequences. From these, fragments of 229, 360 and 679-nt of the right end of IS*801* ended in a consensus tetranucleotide and likely resulted from one-ended transposition of IS*801*. An average 0.7% of the insertions analyzed consisted of IS*801* carrying a fragment of variable size from gene PSPPH_0008/PSPPH_0017, showing that IS*801* can mobilize DNA *in vivo*. Retrospective analysis of complete plasmids and genomes of *P. syringae* suggests, however, that most fragments of IS*801* are likely the result of reorganizations rather than one-ended transpositions, and that this element might preferentially contribute to genome flexibility by generating homologous regions of recombination. A further miniature sequence previously found to affect host range specificity and virulence, designated MITE*Psy1* (100-nt), represented an average 2.4% of the total number of insertions entrapped in *sacB*, demonstrating for the first time the mobilization of a MITE in bacteria.

## Introduction

Insertion sequences (IS) are probably the simplest autonomous mobile DNA elements and generally consist of a transposase coding gene, responsible for their transposition, which is bound by terminal inverted repeats [1], [2]. ISs can generate significant variability in bacteria and contribute to their evolution [3], in part because they are usually present in more than one copy per genome and thus represent mobile regions of recombination. Their mobility, together with their capacity to mobilize unrelated DNA in their proximity, can lead to a panoply of mutations and rearrangements in the host bacteria, which include insertions, deletions, duplications, translocations, cointegrations, inversions and gene activation [4]. From these activities, it easily follows that they have an enormous potential to alter the genome and influence bacterial evolution. They can also shuffle DNA among different genetic replicons such as chromosomes and plasmids sustaining a gene trading activity that widely contributes to the horizontal spread of genetic information [e.g. 5], [6]. ISs are widespread among bacteria and archaea, present in nearly all of the sequenced genomes and often in high numbers [1]. There is also a large variety of ISs, with more than 2500 types included in the IS repository database and grouped in 25 families [7]. Additional types of small, non-autonomous mobile sequences are the REPINs (repetitive extragenic palindromic (REP) doublets forming hairpins) [8] and MITEs (miniature inverted-repeat transposable elements) [1]. MITEs are generally less than 300 bp long and usually contain terminal inverted repeat sequences; MITEs are thought to derive from ISs by internal deletions and to be mobilized *in trans* by the transposase of their parental IS [1], [9]. The impact of MITE activity in the prokaryotic genome is potentially very high and their small size allows them to contribute to phenotypic variation in many different and creative ways, such as generating new gene alleles and functions, or new regulatory signals for preexisting genes [9].

*Pseudomonas syringae* is a plant pathogenic gamma proteobacterium that is being extensively used as a model to study the molecular bases of plant-microbe interactions and the evolution of pathogenicity [10]. In this respect, *P. syringae* is a major study subject because of the large pathogenic variation within the species. Indeed, *P. syringae* can be divided into at least 60 groups, or pathovars, that are characterized by their host range [11]. While the different pathovars show a highly conserved core genome, there is a large variation in their virulence gene complement [12], [13]. The pathogenicity of *P. syringae* depends on the activity of a type III secretion system that delivers specialized proteins, known as effectors, into the plant cell, where they contribute to the suppression of the plant defense responses and the establishment of an infection [10]. Occasionally, effectors are recognized by the plant machinery, leading to the activation of a general defense response, the hypersensitive response, which ultimately leads to a complete plant resistance phenotype. Therefore, effectors might have a dual effect, either promoting pathogenicity and virulence or restricting host range in specific plants. Remarkably, effectors and other virulence genes are commonly bordered by ISs and other types of repeated elements in diverse strains of *P. syringae* [14], [15], [16], which would likely favor their frequent horizontal transfer and exchange. This is supported by the fact that mobile genetic elements are often associated with regions that are not syntenic among different *P. syringae* genomes [17], [18], [19]. Sequencing of native plasmids and genomes has revealed a diverse collection of ISs in pathovars of *P. syringae* [17], [19], [20], [21], [22], with partial or complete sequences of 32 newly described *P. syringae* ISs included in the IS Finder repository. However, it is not possible to estimate the actual diversity due to the lack of a complete IS inventory and the use of different names for the same element. Additionally, there is a large variation in the types and frequency of ISs in the only three complete genomes currently available, of *P. syringae* pv. tomato DC3000 [19], *P. syringae* pv. phaseolicola 1448A [17] and *P. syringae* pv. syringae B728a [21]. Nevertheless, the ability to transpose within *P. syringae* has been demonstrated for only a few elements, including IS*51* (syn. IS*Psy21*) and IS*52* [23], IS*801* [24], IS*Psy2* and IS*Psy3* [25], and the *hopX1* effector transposon [26].

*P. syringae* pv. phaseolicola is the causal agent of halo blight of beans (*Phaseolus vulgaris* L.) and is used as a model for the study of the molecular basis of pathogenicity and virulence [27]. We are interested in the characterization of transposable elements in this bacterium in order to explore their impact on the evolution of virulence and in the frequent genome changes that were reported to occur during the interaction with plant hosts [28]. Additionally, and due to the current lack of other suitable markers, we want to expand the use of repeated DNA for typing populations [29], [30]. Repeated DNA has been directly involved in shaping host range in *P. syringae* pv. phaseolicola by the inactivation or alteration of effector genes [31], [32]. A chimeric transposable element promoted large deletions eliminating effector gene *avrPphF* (syn. *hopF1*), allowing the emergence of populations that overcame the resistance conferred by gene *R1*, which was widely used for the control of halo blight [32]. Likewise, the generation of new alleles of effector gene *avrPphE* (syn. *hopX1*) after the insertion of a small repeated sequence [31], which has been determined in this work to be a MITE (see below), lead to the emergence of new pathogenic races. Genome rearrangements have also been linked to the activity of repeated DNA. Originally isolated from a *P. syringae* pv. phaseolicola strain, IS*801* was shown to participate in the integration and excision of a native plasmid mediated by recombination between copies of the IS, which resulted in a dynamic exchange of DNA between the plasmid and the chromosome [24], [33]. IS*801* belongs to the IS*91* family of rolling circle transposable elements, which seem to be preferentially involved in the dissemination of pathogenicity-related genes [34], [35], [36]. Nevertheless, there is still limited information on the content and functionality of transposable elements in this pathogen. Five percent of the ORFs in the closed genome of *P. syringae* pv. phaseolicola 1448A were identified as mobile genetic elements [17], but this figure includes integrases and phage-related DNA sequences, making it difficult to estimate the relevance of transposable elements. Although an inventory of transposable elements in strain 1448A is not available, the annotation of the genome (accession no. CP000058) includes the coding regions for transposases, but not other non-coding sequences related to transposable elements, limiting the utility of this information.

To characterize and quantify the impact of transposable elements on the life cycle of *P. syringae*, we analyzed and reannotated the genome of the model bacterium *P. syringae* pv. phaseolicola 1448A. We also used an IS trapping vector to identify functionally active elements and assess their impact in the genome flexibility. This study provides a foundation for a larger scale analysis of transposable elements in other *P. syringae* genomes.

## Results

### *P. syringae* pv. phaseolicola 1448A contains at least seventeen insertion sequences and two MITEs

A comparison of the genome of *P. syringae* pv. phaseolicola 1448A with the databases indicates that it harbors at least seventeen different types of insertion sequences and two MITEs (Table 1 and Annotation files S1, S2 and S3). The transposases of these insertion sequences have all been previously annotated in the genome of strain 1448A [17], and here we contributed the definition and annotation of the complete elements, as well as the identification of 21 truncated copies and several MITEs that were not annotated previously.

**Table 1:** Type and number of mobile elements found in the genome of *P. syringae* pv. phaseolicola 1448A^a^.

						Number of insertions		
Mobile element^b^	Synonym	Family	Size (nt)	No. of CDSs	Inverted repeats	Chromosome	pA	pB
IS*51*	IS*Psy21*	IS*3*	1312	2	26	2	1 (1)	-
IS*53*	IS*Psy20*	IS*21*	2572	2	27	2 (3)	-	-
IS*801*		IS*91*	1512	1	no	3 (6)	1 (10)	-
IS*Psy2*		IS*5*	1194	1	12	5 (6)	(2)	-
IS*Psy3*		IS*91*	1507	1	no	-	(3)	-
IS*Psy4*	IS*Psy23*	IS*21*	1962	2	23	(3)	(1)	-
IS*Psy16*		IS*110*	1461	1	12	2 (1)	3 (3)	-
IS*Psy17*	IS*Psy18*	IS*256*	1374	1	28	47 (10)	(4)	1
IS*Psy19*		IS*5*	1178	1	17	28 (10)	3	-
IS*Psy22*		IS*5*	unk	unk	unk	(2)	-	-
IS*Psy24*		IS*3*	1235	2	26	2 (4)	(1)	-
IS*Psy25*		IS*630*	≥1177	1	19	1(1)	-	-
IS*Psy26*	IS*Psy29*	IS*3*	≥1231	2	28	-	1 (1)	-
unnamed^c^		unk	unk	unk	unk	(1)	-	-
unnamed^d^		Tn3	unk	unk	unk	(1)	(2)	-
unnamed^e^		IS*66*	unk	unk	unk	(1)	-	-
unnamed^f^		IS*66*	unk	unk	unk	(1)	-	-
MITE*Psy1*		IS5?	100	0	18	5	1	-
MITE*Psy2*		unk	228	0	26	1	1	-

a Only repeats larger than 200 bp are included in the Table, except in the case of the MITE*Psy1* element. No. of CDSs indicates the number of coding sequences found in the mobile element; inverted repeats indicate the number of nucleotides in each terminal inverted repeat. Numbers in parentheses indicate degenerate elements. unk, unknown; -, indicates the element was not detected.

b The following insertion elements, previously found in *P. syringae*, were not present in 1448A: IS*Ps1*, IS*Pssy*, IS*Psy1*, IS*Psy5* IS*Psy6*, IS*Psy7*, IS*Psy8*, IS*Psy9*, IS*Psy10*, IS*Psy11*, IS*Psy12*, IS*Psy13*, IS*Psy14*, IS*Psy15*, IS*Psy27*, IS*Psy28*, IS*Psy30*, IS*52*, and IS*1240*. The size of IS*Psy25* and IS*Psy26* was estimated to be at least the size of the transposase plus the surrounding DNA that included the typical bordering inverted repeats.

c Correspond to loci PSPPH_0182-PSPPH_0183. Loci PSPPH_0182 belongs to family Pfam PF05621, of NTP-binding proteins involved in transposition. PSPPH_0183 is reorganized, but contains a C-terminal Mu transposase domain (PF09299) found in various prokaryotic integrases and transposases.

d Correspond to loci PSPPH_3494, PSPPHA0085, and PSPPHA0131.

e Corresponds to locus PSPPH_3497.

f Corresponds to locus PSPPH_4298.

The copy number of transposable elements was highly variable, from one to 48 complete copies, and many of them were fragmented, suggesting the occurrence of DNA reorganizations. In total, the mobile DNA amounted to at least 199,018 nt, representing 3.3% of the nearly 6 Mb genome (Table 2); however, the density of ISs varied for the chromosome (2.8%), the 132 kb plasmid (25.8%), and the 52 kb plasmid (2.7%). Coding sequences (CDSs) for transposases were reported to clearly disrupt 8 reading frames in the 1448A genome (Table S1) [17]. Our annotation of the complete mobile elements reveals that they interrupt at least five other CDSs and form chimeras with at least 40 CDSs (Table S1). The majority of these CDSs would likely have an altered functionality or not be functional at all, as occurs with the interrupted CDSs and 11 chimeric CDSs whose 5′ end correspond to mobile DNA sequences.

**Table 2:** Amount of DNA corresponding to putative insertion sequences in the genome of *P. syringae* pv. phaseolicola 1448A.

	Size (kb)	Insertion sequence DNA (kb)	% of ISs
Chromosome	5928	163.6	2.8
p1448A-A	132	34.0	25.8
p1448A-B	52	1.4	2.7
Total	6112	199.0	3.3

Nine copies of IS*801* (copies IS*801*.T1 to T6 and T10 to T12) and three copies of IS*Psy19* (IS*Psy19*.T6 to T8), were of wild type length but contained a premature stop in the transposase gene. Since the IS*801* transposase can efficiently act in *trans* [36], it is conceivable that the interrupted derivatives of IS*801* could also transpose in the cell. Element IS*Psy17*.T5 is also full-length but interrupted by a tandem insertion of IS*Psy19* and of a new mobile element identified in this work and designated MITE*Psy1* (see below). Additionally, element IS*Psy17*.38 contains an independent insertion of MITE*Psy1* 14 nt upstream of the transposase start codon, although this would predictably not affect transposition of the element because it does not affect the terminal repeats or the CDS.

There is a very limited description of insertion sequences IS*Psy17*, IS*Psy19* and IS*Psy24*, for which nearly only the transposase genes were identified [17], [32]. Therefore, to estimate the size of the complete element, we aligned the coding sequences of the corresponding transposases found in the genome of 1448A plus up to 1 kb on either side. The longest consensus sequence that contained terminal repeats, but not any duplicated sequence resulting from insertion, was considered to be that of the full-length insertion sequence.

For IS*Psy17*, we determined a consensus sequence of 1374 nt that was bordered by 28 nt imperfect repeats (Tables 1 and 3). The right border defined in this way coincided with that previously defined from deletion variants generated by a chimeric element derived from IS*Psy17* [32]. The element includes a 1260 nt ORF that could code for a putative 419 aa transposase, which was found in 48 intact copies and 62 in total, in the genome of *P. syringae* pv. phaseolicola 1448A (Table 1). The predicted start codon of the transposase is located only 53 nt downstream of the 5′ end of the element, which may indicate that transcription of the transposase gene could be exclusively under the control of promoters upstream of the insertion point (*i.e.* promoters outside of the IS). We analyzed a comparison of the 1448A genome with those of *P. syringae* pv. syringae B728a and *P. syringae* pv. tomato DC3000, which do not harbor IS*Psy17*. Only 13 of the 47 chromosomal insertions of IS*Psy17* (insertions 1, 2, 4, 8, 10, 12, 16, 19, 27, 37–39, 45) in 1448A were located in DNA regions that were otherwise complete in at least one of the two other bacteria. These insertions, that we will call here “genuine insertions”, were surrounded in 10 out of 13 cases by a perfect target duplication of 6 or 8 nt. In all, twenty four insertions were surrounded by an 8 nt target duplication, whereas others were surrounded by either a 6 nt duplication, an imperfect 4 to 5 nt direct repeat or by no discernible target repeat (Table 4). Although in all cases the sequence was apparently unique for each of the target duplications, we evaluated the possible occurrence of a target preference for IS*Psy17*. For this, we constructed alignments of all the insertions with a perfect 8 nt duplication, because members of the IS*256* family, such as IS*Psy17*, generate an 8 or 9 bp direct target repeat [2]. From these alignments, we could not discern any obvious consensus sequence that could serve as the insertion target, except that there was always a C or a T 24 nt downstream of the 3′ end of IS*Psy17*, suggesting that this element might insert randomly.

**Table 3:** Characteristics of selected mobile elements in *P. syringae* pv. phaseolicola 1448A.

Insertion element	Terminal inverted repeats^a^	Sequence duplication^b^	Target sequence^b^
IS*Psy17*	GAGACTGTCAGAAATTTTGTGTTCGGGC |.||.|.||||…|||||||||||||| GGGAGTATCAGTTTTTTTGTGTTCGGGC	Probably 8 nt	Random
IS*Psy19*	GAGGGTGTAGACAAAAT ||||||||||||||||| GAGGGTGTAGACAAAAT	3 nt	CHHD
IS*Psy24*	TGTAGTGGTCTAATGAAACCGGACAC ||||||||||.|.|||..|||||||| TGTAGTGGTCAACTGATCCCGGACAC	4 nt ?	unk
MITE*Psy1*	GGAAGGTCTGAAAAAGCC |.|…|||||||||||| GTATCCTCTGAAAAAGCC	4 nt	CTAG or YTAA
MITE*Psy2*	GGGGGTGTAAGCCAGAACCGCCGAAAATTCCGTC ||||.||||||||.||||||||||||.||||||| GGGGTTGTAAGCCGGAACCGCCGAAATTTCCGTC	unk	unk

a Terminal inverted repeats are shown 5′-3′, with the left repeat in the top and the right repeat at the bottom.

b unk, unknown.

**Table 4:** Nucleotide direct repeats found bordering insertions of IS*Psy17* in *P. syringae* pv. phaseolicola 1448A^a^.

	Type of insertion	
Direct repeat	Genuine	Other
Perfect 8 nt	7	13
Imperfect 8 nt^b^	3	1
7 nt	-	3
6 nt	3	1
Degenerate^c^	-	5
None	-	12

a Only those insertions containing a complete copy of IS*Psy17* were analyzed. Genuine insertions are those that disrupt a sequence in 1448A that in genome comparisons is continuous in the genomes of *P. syringae* pv. syringae B728a or *P. syringae* pv. *tomato* DC3000.

b These insertions consist of an 8 nt direct repeat with 1–2 mismatches or a 1 nt insertion.

c Degenerate indicates direct repeats of 4–5 nt, which might be surrounded by mismatches.

IS*Psy19* was defined as a 1178 nt element containing a perfect 17 nt inverted repeat (Tables 1 and 3) and coding for a putative 367 aa transposase. There were 31 intact copies and it was often associated with other mobile elements, with 9 complete or degenerate insertions adjacent to IS*Psy2* and three adjacent to MITE*Psy1*. Six chromosomal insertions of IS*Psy19* (insertions 7, 10, 14, 18, 27, and T7) could be considered genuine insertions in a genome comparison with strains B728a and DC3000. Fifteen of the IS*Psy19* copies were surrounded by a 3 nt perfect duplication of the target DNA; alignment of the DNA surrounding these elements suggests that the insertion target for IS*Psy19* is the sequence CHHD.

IS*Psy24* is a 1235 nt element surrounded by an imperfect 26 nt repeat (Table 3) and coding for two putative proteins of 102 and 273 aa, of which the first belongs to the IS*3* family of tranposases (InterPro IPR002514) whereas the second contains a ribonuclease H-like domain (InterPro IPR012337). IS*Psy24* is present only in two intact copies, and only one of them generated a 4 nt target repeat; therefore, it is not possible to deduce if this element has any target preference or if it leads to target duplication.

**Figure 1:**
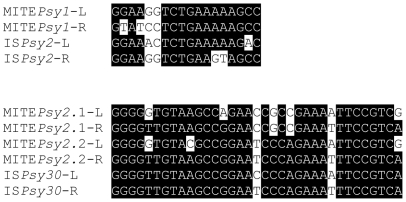
Conservation of the terminal repeats of MITE*Psy1* and MITE*Psy2*. A black background indicates conservation of each nucleotide in at least three quarters of the sequences. The ends of the six copies of MITE*Psy1* in strain 1448A are identical; for MITE*Psy2*, the ends of the chromosomal copy (MITE*Psy2*.1) and of the copy in plasmid p1448A-A (MITE*Psy2*.2) are shown.

A 104 nt sequence with terminal inverted repeats was previously found inserted into effector gene *avrPphE* (syn. *hopX1*) in race 8 strains of *P. syringae* pv. phaseolicola, leading to a change in host range [31]. We found that this sequence is present in several copies in the 1448A genome (Table 1) and was able to transpose (see below); we therefore considered it to be a functional MITE and designated it as MITE*Psy1*. Comparison of the MITE*Psy1* insertions in 1448A and in other *P. syringae* pv. phaseolicola strains (accession no. AB023077 and AY147025), plus the analysis of the insertions entrapped with pGEN500 (Table 5), indicate that this element is 100 nt long, has 18 nt terminal inverted repeats and that it inserts after the sequence CTAG or YTAA, which is duplicated upon insertion (Table 3). The high similarity between their repeated sequences suggests that MITE*Psy1* might have generated from IS*Psy2* (Figure 1). BlastN comparisons with microbial genomes indicated that well conserved (>97% identity) copies of MITE*Psy1* are present only in *P. syringae* pvs aesculi 0893-23 and NCPPB 3681, glycinea B076, lachrymans M301315, mori 301020 and savastanoi NCPPB 3335, all belonging to genomospecies 2. Additional sequences homologous to MITE*Psy1* were only found in the genomes of *P. syringae* pvs actinidiae M302091 (91 nt, 77% identity) and tomato DC3000 (52 nt, 91% identity) (Figure S1), indicating a somewhat restricted distribution of this element in the *P. syringae* complex. Additionally, the genomes of *P. stutzeri* ATCC 17588 and of the denitrifying and alkane degrading *Gammaproteobacterium* HdN1 (accession no. FP929140) contain an identical 99 nt sequence that showed a global 71% identity with MITE*Psy1* (Figure S1). A further Blast search with the homolog from *P. stutzeri* found similar sequences in plasmids or chromosomes of diverse *Pseudomonas* species, *Marinobacter aquaeolei*, *Klebsiella pneumoniae* and plasmid pRSB105 from an uncultured bacterium, suggesting that sequences similar to MITE*Psy1* might be widespread among bacteria.

**Table 5:** Type and number of mobile elements entrapped in three populations of transformants (PT) and four individual transformants (T1 to T4) of *P. syringae* pv. phaseolicola 1448A containing the entrapment vector pGEN500.

		Number of plasmids carrying a mobile element									
Mobile element	Size (kb)	PT1	PT2	PT3	T1	T2	T3	T4	Total	% of suc^R^ plasmids	% of insertions^a^
Transposition frequency (×10^−6^)		26.0±3.0	3.7±1.2	3.8±0.6	13.0±2.4	1.1±0.5	9.8±0.8	9.6±0.8			
IS*801*	>1.5	1	0	0	1	0	0	1	3	0.7	0.7
	1.5	69	62	56	25	30	31	27	300	65.2	71.2
	0.679	19	13	25	6	4	1	3	71	15.4	16.9
	0.360	0	0	0	0	1	0	1	2	0.4	0.5
	0.229	6	15	8	2	0	3	1	35	7.6	8.3
MITE*Psy1*	0.1	1	0	4	1	1	1	2	10	2.2	2.4
None	-	4	9	8	5	4	4	5	39	8.5	
Total no.		100	99	101	40	40	40	40	460		

a Average percentage of each insertion type over the total number of insertions on *sacB*.

A putative 228 nt MITE, designated MITE*Psy2*, is present in the native plasmids of *P. syringae* pv. *savastanoi* NCPPB 3335 [37]. This element contains terminal repeats highly similar to those of IS*Psy30*, an insertion sequence of the Tn*3* family also present in strain NCPPB 3335 and in several other *P. syringae* strains (Figure 1). MITE*Psy2* is also present in two copies in the 1448A genome (Table 1), and shows 26 nt imperfect inverted repeats (Table 3). Homologs of this MITE are widespread in the *P. syringae* group, although it shows high sequence variability with identity levels in pair comparisons as low as 78%.

Importantly, neither MITE*Psy1* nor MITE*Psy2* were identified using the programs MUST [38] and MITE-Hunter [Han, personal communication; 39], specifically designed for the detection of MITEs, indicating the need to design enhanced prediction programs.

### The transposition frequency is similar in different growing conditions

Our analysis suggested that there have been unique, independent transposition events within 1448A. To test the hypothesis that some of these transposons are transposing within the genome, we estimated transposition frequency of mobile DNA in *P. syringae* pv. phaseolicola using the entrapment vector pGEN500 [40]. This plasmid contains gene *sacB* from *Bacillus subtilis*, which confers sucrose-dependent lethality to many Gram-negative bacteria, thus providing a positive selection for insertions of mobile elements into *sacB* [41]. We examined transposition using individual transformants of *P. syringae* pv. phaseolicola 1448A as well as heterogeneous bacterial populations originated from single transformation experiments, which we designated as “pool of transformants”.

The overall transposition frequency of different pools of transformants or clones of *P. syringae* pv. phaseolicola 1448A(pGEN500), originating from independent electroporation events, ranged from 2.6×10^−5^ to 1.1×10^−6^, depending on the transformant (Table 5). Similar frequencies were observed after six consecutive rounds of growth in liquid KMB plus tetracycline (not shown), suggesting that suc^R^ clones did not accumulate in the bacterial populations. We found comparable transposition frequencies using *P. syringae* pv. phaseolicola strain 1449B (race 7; not shown), suggesting that the data obtained with strain 1448A might be representative of this pathovar. Since there is a similar proportion of the different types of insertion among the different transformants analyzed (Table 5), this variation must be due to phenomena that have a general effect. An obvious explanation is that, for each transformant, there is a differential level of toxicity of the entrapment vector in the absence of sucrose. This would predictably cause the premature death of cells, even though they are not exposed to sucrose, and would result in an artificial increase of the apparent rate of transposition. Indeed, in some clones we observed a lack of correspondence between the optical density of the culture and the expected number of colony forming units (not shown). If this would be the case, then the use of entrapment vectors based on *sacB* would not allow for the estimation of accurate absolute transposition frequencies, at least in *P. syringae*, although it could be used for comparative analyses.

Transposition activity has been reported to be influenced by the growth environment [42], and particularly by diverse stressful conditions [40], [43], [44], although the effect of host and growth factors on transposition are considered to be specific for each mobile element [2], [42]. We therefore evaluated the transposition frequency in bacteria subjected to both favorable and stressful conditions using five independent transformants of strain 1448A(pGEN500). For all the conditions tested, there were no significant differences among the frequencies estimated independently for each of the transformants and, in consequence, further analyses were done using the combined data of all the transformants for each condition. The average transformation frequency was not significantly different for any of the conditions tested, which included bacteria grown in liquid rich medium (KMB; transposition frequency 7.72±6.85×10^−6^) or minimal medium (MG; 6.56±5.73×10^−6^), and bacteria recovered from artificially inoculated bean cv. Tendergreen (compatible host; 6.77±3.51×10^−6^) and tobacco leaves (incompatible host; 3.87±2.31×10^−6^). These results suggest that growth conditions do not have a significant influence on the transposition frequency of mobile DNA in *P. syringae* pv. phaseolicola 1448A.

### Only two mobile elements were identified in a functional assay using an entrapment vector

We were able to use the strains recovered from the previous experiments to identify the mobile DNA disrupting *sacB* in pGEN500. A preliminary sequence analysis of over 40 clones indicated that pGEN500 contained insertions of IS*801* in nearly all the suc^R^ colonies. Therefore, native plasmid profiles from 460 suc^R^ colonies obtained from three independent pools of transformants and four individual transformants were analyzed by Southern hybridization using a specific IS*801* probe. Only 10.7% of the plasmids in suc^R^ clones did not hybridize with IS*801* (Table 5). Sequencing of the *sacB* gene in these strains revealed that they either contained insertions of MITE*Psy1* (2.2% of the plasmids) or had deletions or point mutations in *sacB* (8.5%). All the insertions of MITE*Psy1* produced, as expected, a 4 nt duplication of the target sequence (Table 3).

**Figure 2:**
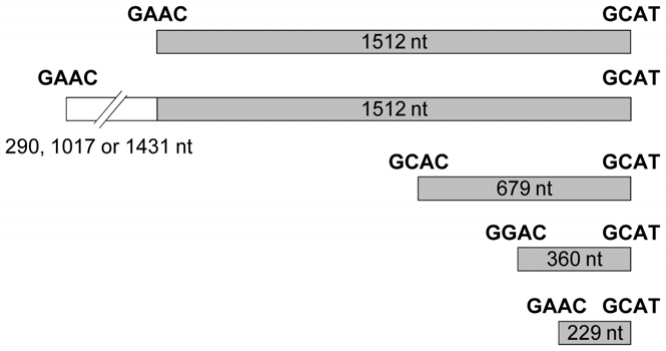
Structure and terminal ends in IS*801* and miniature derivatives found to actively transpose in *P. syringae* pv. phaseolicola 1448A. Grey boxes indicate the wild type IS*801* (1512 nt) or miniature sequences derived from the complete element, with their size indicated inside the box; all of them share the same right terminus and continue without gaps as indicated by their relative position. The sequences indicate the tetranucleotides marking their left and right ends. The broken white box indicates the three different fragments of gene PSPPH_008/PSPPH_0017 that were mobilized by IS*801* in three different experiments, with an indication of their sizes. Drawings are to scale.

The remaining pGEN500 derivatives, representing 89.3%, contained DNA cross-hybridizing to the IS*801* probe (Table 5), although the size of the insertions was variable. Only 65% of these plasmids contained inserts of a size compatible with the full-length IS*801* (1512 nt), whereas the remaining plasmids contained inserts derived from IS*801* of 229 nt, 360 nt, 679 nt, and over 1.5 Kb (Figure 2), as shown by DNA sequencing. The 229, 360 and 679 nt derivatives, representing 26.3% of the total number of IS*801* insertions, are the result of a one-ended transposition and consist of continuous fragments flanked by the right terminal repeat and by a tetranucleotide with homology to the wild type left end of IS*801* (Figure 2). The larger fragments consisted of a complete copy of IS*801* that had recruited 290, 1017 or 1431 nt of the DNA immediately upstream of the element (Figure 2), ending in a tetranucleotide identical to the 5′ end of IS*801*. The mobilized DNA and the preceding IS*801* copy are duplicated with 100% identity in the genome [PSPPH_0007 (IS*801*) and PSPPH_0008 (oxidoreductase); PSPPH_0016 (IS*801*) and PSPPH_0017 (oxidoreductase)], and therefore we were unable to discern if they correspond to the first (PSPPH_0007 and PSPPH_0008) or to the second copy (PSPPH_0016 and PSPPH_0017).

Partial fragments of IS*801* are very often associated to virulence genes [14], [34], so we questioned if they could have originated from one-ended transposition events. In a Blast comparison, IS*801* was present in only a portion of the closed genomes and plasmids of *P. syringae* strains, where we found 17 truncated fragments (Table 6), of which 11 were inserted less than 5 kb away from a putative virulence gene. Only five of these 17 fragments contained an intact right repeat and lacked the left repeat. However, only two of the five fragments could be considered as the result of a one-ended transposition because their 5′ end was homologous to the left repeat of IS*801*; both fragments were identical to the 229 nt functional fragment identified in this work. The analysis of partial sequences from diverse *P. syringae* strains previously described to contain IS*801* fragments [34] also showed that none of them had the typical characteristics of miniature fragments originating from one-ended transposition (containing an intact right end repeat and having as a left end a tetranucleotide with homology to the wild type left end of IS*801*). This suggests that the large majority of IS*801* fragments in the *P. syringae* genomes, and associated to virulence genes, is probably the result of DNA reorganizations.

**Table 6:** Type of truncated fragments derived from IS*801* found in closed genomes of *Pseudomonas syringae*.^a^

			Number of each type of IS*801* truncation					
Genome	Molecule	accession no.	5′	Internal	3′	Both ends	Total no.	Possible mobile fragment^b^
*P. syringae* pv. phaseolicola 1448A	Chromosome	CP000058	-	-	1	-	1	-
	p1448A-A	CP000059	3	1	2	1	7	2 (229 nt)
*P. syringae* pv. maculicola ES4326	pPMA4326B	AY603980	-	2	1	-	3	-
*P. syringae* pv. tomato DC3000^c^	Chromosome	AE016853	-	1	-	1	2	-
	pDC3000A	AE016855	1	-	-	-	1	-
	pDC3000B	AE016854	1	1	1	-	3	-

a Only truncated fragments larger than 100 nt were taken into account, but not the complete element with premature stops; (-) indicates that no fragments were found in the category. Plasmid pFKN (accession no. AF359557) contains a copy of IS*801*, but not truncated forms. The following sequences (accession no.) did not contain any IS*801* sequences: p1448A-B (CP000060), pPMA4326A (AY603979), pPMA4326C (AY603982), pPMA4326D (AY603981), pPMA4326E (AY603983), pPSR1 (AY342395), and *P. syringae* pv. syringae B728a (CP000075).

b We assumed that, as it happens with IS*91* [34], the right end (3′ end) of IS*801* is essential for transposition; therefore, partial fragments of IS*801* were considered to be able to transpose when: (1) they contained an intact right end, and (2) the fragment started with any of the tetranucleotides described as an IS*801* insertion target (GAAC, GGAC, CAAG and CGAC) [36]; these partial fragments could be found only among those with a 5′ end deletion because fragments with internal deletions were specifically excluded.

c IS*801* has not been described in strain DC3000 [19], and the fragments found in this strain probably correspond to insertion sequence IS*Psy3*, a close relative of IS*801* that also belongs to the IS*91* family.

### Transpositions do not occur in bursts

Transposition could occur in the cell either as a single event or as multiple simultaneous events. To investigate this in *P. syringae*, we examined the hybridization pattern of five clones of *P. syringae* pv. phaseolicola 1448A(pGEN500), each containing a different type of insertion in *sacB* (see Table 5), using insertion sequences IS*53* (syn. IS*Psy20*), IS*801*, IS*Psy2*, and IS*Psy24* as probes. All the clones showed identical patterns of hybridization with all the probes, with the exception of the bands corresponding to the insertion in the entrapment plasmid, suggesting that a single insertion has occurred in each of them (Figure S2 and data not shown).

## Discussion

The very large variability in the occurrence of ISs in prokaryotic genomes cannot be easily explained and, unexpectedly, the analysis of over 200 genomes indicated that the variability is apparently not correlated with pathogenicity or rates of horizontal gene transfer, with genome size being the only significant predictor of abundance and diversity [45]. The genome of *P. syringae* pv. phaseolicola 1448A contains 102 complete IS elements, which is nearly twice the number that would be expected given its genome size [45]. In this case, it is likely that the complement of virulence genes present in strain 1448A is partly responsible for this abundance, given the close association between virulence genes and mobile elements in *P. syringae*, and in particular with IS*801* [14], [34], [46].

There is a remarkable asymmetry in the distribution of mobile elements in *P. syringae* pv. phaseolicola 1448A, representing over a quarter of the large 132 kb plasmid (p1448A-A) but only under 3% of the 52 kb small plasmid (p1448A-B). Since the percentage of IS DNA in plasmids larger than 20 kb averages 5–15%, reaching 20% in some cases [1], these figures are also outside of the range normally found in other plasmids of equivalent size. This asymmetry is also found in the native plasmids of *P. syringae* pv. savastanoi NCPPB 3335, where less than 4% of the 45 kb plasmid pPsv48B corresponds to mobile DNA, whereas they represent nearly a quarter and a third of the size for the 78 and the 42 kb plasmids, respectively [37]. We do not yet have an explanation for this asymmetry, which strongly disagrees with the observation that genome size could predict IS abundance [45], and that cannot be easily justified based on the gene content of these plasmids. Both p1448A-B and pPsv48B appear to mostly contain genes for their survival with a very low number of potential virulence genes [17], [37]. By contrast, p1448A-A carries several virulence genes and a pathogenicity island that is essential to produce disease in the plant host, bean [16], [17]. Since genes are generally inactivated upon IS insertion, we may expect a higher number of insertions of mobile elements in the predictably dispensable plasmid p1448A-B, rather than in the essential virulence plasmid, p1448A-A. Nevertheless, p1448A-B contains a whole type IV secretion system, spanning around 25 kb, which is also present in pPsv48B and that might be implicated in plasmid survival or virulence [47], [48]. It is therefore possible that evolution selects against insertions in p1448A-B and pPsv48B, at least in part, because they could compromise the integrity of the type IV system.

Using an entrapment vector in a functional assay, we identified the mobilization of only IS*801* and MITE*Psy1*, in spite that *P. syringae* pv. phaseolicola 1448A harbors at least nineteen putative types of mobile elements. The number of intact copies of these two elements in the genome of strain 1448A is relative low, 4 and 6 copies, respectively, compared to the 48 copies of IS*Psy17* or the 31 of IS*Psy19*, suggesting that insertions of these elements do not accumulate to high numbers. In particular, the number of insertions of IS*801* do not appear to be very different in comparisons between and within the two genetic lineages previously defined in *P. syringae* pv. phaseolicola [29]. Remarkably, we observed the transposition of miniature sequences corresponding to partial fragments of IS*801* (see below) that, together with MITE*Psy1* represented around 28% of the total mobilized elements in strain 1448A. We did not observe the transposition of any of the other mobile elements found in strain 1448A among the ca. 500 clones analyzed, indicating that they might transpose at a frequency lower than 10^−8^, that they have become fixed in the chromosome or that other conditions stimulate excision. Given their apparent stability, these mobile elements might therefore be suitable as epidemiological markers. The transposition frequency was stable for each clone in all growing conditions examined, including repeated transfers in culture, suggesting that the frequency of transposition in *P. syringae* pv. phaseolicola is not influenced by the type of interaction with the plant host or by the growing medium. Nevertheless, we cannot rule out that the different growing conditions are affecting the relative proportion of each type of mobile element that are transposing, or their insertion specificity.

Members of the IS*91* family, including IS*801*, are evolutionary close to diverse plasmids and single-stranded phages, such as ΦX174 and, unlike other mobile elements, are thought to transpose by a rolling circle replicative mechanism, producing permanent insertions and efficiently transposing with a transposase provided *in trans* [34], [35], [36]. IS*91* lacks proper terminal inverted repeats and its transposition is thus similar to a round of plasmid replication. Transposition of IS*91* originates at the right terminal repeat, which is called *ori91* and is essential for this process [49], and ends in the left repeat, called *ter91*, or in any other sequences with homology with *ter91*, resulting in one-ended transposition [34], [49]. IS*801* was suspected to also undergo one-ended transposition [36], and we demonstrate here that it occurs with a very high frequency in its natural bacterial host, representing more than 26% of the total IS*801* transposition events (Table 5). The left end of members of the IS*91* family shows a highly conserved 17 nt G-C rich sequence that includes a potential stem and loop structure [35]. However, we did not find this sequence in the 5′ end of any of the three miniature fragments identified here, suggesting that it might be irrelevant for transposition. Likewise, we did not identify in these ends any obvious repeat, palindrome or consensus sequence, except for the conserved tetranucleotide marking the left end of the fragment (see Figure 2). This suggests that any IS*801* fragment could move in one-ended transposition event as long as it contains the right IS*801* end and any of the tetranucleotides with homology to the left end of IS*801* (GAAC). However, in the original sequence of IS*801* (accession no. X57269) there are 7 occurrences of the tetranucleotides GAAC, GGAC, CAAG and CGAC, plus 11 of GCAC; these sequences border the IS*801* miniature fragments (Figure 2) and were previously identified as target sequences for IS*801* insertion [36], except GCAC. In spite of this, we observed one-ended transposition of only three of these 18 potential transposing fragments. Additionally, although the tetranucleotide GCAC found at the beginning of the 679 nt fragment has not been described as a target sequence for IS*801*, this fragment was mobilized with a much higher frequency than the others (Table 5). Therefore, it is likely that other sequences around the left end might be important for recognition of the IS*801* left terminus and complete the transposition process. Importantly, the miniature IS*801* derivatives identified here must be the result of the partial transposition of full-length elements, rather than the mobilization of pre-existing submolecular fragments, because there are no copies of the 679 or 360 nt fragments in the 1448A genome. This clearly indicates that transposition of a complete IS*801* copy is normally inefficient, producing the mobilization of miniature fragments with a high frequency.

As occurs with IS*91* [34], IS*801* was also capable of mobilizing adjacent DNA, albeit at a very low frequency. In three independent events, we observed the mobilization of different partial fragments of the same gene, PSPPH_0008/PSPPH_0017 coding for the alpha subunit of a molybdopterin oxidoreductase, which in all cases started in the same tetranucleotide than the IS*801* left end, GAAC (Figure 1). We do not have a satisfactory explanation for this preference; moreover, both PSPPH_0008 and PSPPH_0017 are probably non-functional because the IS*801* insertion appears to have eliminated most of the 3′ end of the gene (Table S1), while at least 153 nt are missing from the 5′ end of the PSPPH_0008 reading frame in comparison with its closest homolog (not shown). Nevertheless, our results indicate that IS*801* could potentially mobilize by transposition the virulence genes with which it is often associated. This could be relevant, because elements related to IS*91* are involved in the mobilization of virtually every class of antibiotic resistance genes [50]. Although the mobilization of adjacent DNA by IS*801* occurred at low frequency in our experimental conditions, it is possible that the interaction with plant hosts provides a highly selective environment favoring the exchange of effector and other virulence genes mobilized by IS*801*.

IS*801* is limited in distribution within *P. syringae*, and is tightly associated to virulence genes [34], very often appearing as a partial element of various sizes and whose origins are unclear. Our analysis of partial fragments in complete plasmid and chromosome genomes of *P. syringae* suggests that most of them originated by recombination, rather than by one-ended transposition (Table 6), although a 229 nt fragment in the large plasmid of strain 1448A is interrupting a copy of IS*Psy3* (PSPPH_A0015), suggesting that it originated by a true transposition event. Nevertheless, it is of course possible that any of these miniature fragments could mobilize accompanying DNA, even if this happens at a low frequency. One-ended transposition was proposed to be the main mechanism for gene propagation mediated by IS*91* [34]; conversely, our results suggest that IS*801*, and partial fragments thereof, might preferentially contribute to the generation of recombination regions around virulence genes instead of serving as carrier elements. Indeed, insertion sequences are known to play a major role in genome flexibility, and variation between isolates of *P. syringae* appears to be due more to recombination than to mutation [51], [52].

A remarkable outcome is the identification of a functionally active MITE in strain 1448A, that we have designated MITE*Psy1*; a second putative MITE, designated MITE*Psy2*, was also discovered in the genome at lower copy number, but this was not trapped in the transposition assay. MITEs are non-autonomous elements, in that they lack a transposase and rely entirely on transposases encoded within other elements existing within the genome, which act *in trans* [53]. The lower copy number of MITE*Psy2* and the fact that it was not captured in the transposition assay may indicate that it is a recent addition to the 1448A genome, which lacks a transposase to mobilise it. MITE elements are widespread in bacteria and there is indirect evidence of their mobility [54], [55]. For example, a MITE-like sequence was shown to transpose as a composite element and mobilized an antibiotic resistance gene [56]. However, this is the first time that a MITE is reported to actively transpose *in vivo*, and at a high frequency, opening the way to functionally test the requirements and molecular mechanisms for their transposition. MITE*Psy1* has probably originated from IS*Psy2*, because their terminal repeats are nearly identical; in any case, it is likely that the IS*Psy2* transposase is responsible for the mobility of MITE*Psy1*, because transposase specificity is generally determined by the sequence of the mobile element terminal repeats [2], [4]. However, a potentially contradicting result is that we observed the frequent mobilization of MITE*Psy1*, reaching nearly 2.5% of the total number of insertions (Table 5), although in no case did we observe the mobilization of IS*Psy2* despite strain 1448A encoding five complete copies of this element. Two of the six copies of MITE*Psy1* present in the genome of 1448A are interrupting two loci, PSPPH_0770 and PSPPH_A0017 (Table S1), whereas another two are intergenic and a further two are inside other transposons. The insertion of MITE*Psy1* into a CDS implies a change in the reading frame, because the element is 100 nt long and produces a 4 nt duplication, potentially leading to gene inactivation or to the generation of new alleles. Indeed, insertion of a 104 nt sequence (identified here as MITE*Psy1* plus the 4 nt duplication) in the 3′ end of effector gene *avrPphE* (syn. *hopX1*) in a strain of *P. syringae* pv. phaseolicola race 8 lead to the generation of a new allele that no longer induced the hypersensitive response in resistant bean cultivars, causing the expansion of its host range [31]. Therefore, it shall be important to screen effector genes in different phytopathogenic bacteria for the presence of repeated sequences that might be surrounding them and could either participate in their mobility or alter their coding sequences, because they could represent new miniature mobile elements. Examples of this type of small repeated sequences have already been found in the chromosome and plasmids of different strains of *P. syringae* [17], [57], whereas two different MITEs were found to alter the host range of *Ralstonia solanacearum* and contribute to the generation of epidemic genotypes of the pathogen [55].

## Materials and Methods

### Bacterial strains and growth conditions

*Escherichia coli* DH5α was routinely grown using LB medium [58] at 37°C and was used for cloning purposes. *Pseudomonas syringae* pv. phaseolicola 1448A [race 6], [; 59] and 1449B [race 7; 60] were routinely propagated at 25°C using King's medium B (KMB) [61] and the frequency of transposition was generally estimated using nutrient agar (NA; Oxoid, Basingstoke, UK) and NA supplemented with 5% (w/v) sucrose (SNA). Medium MG [62] was used as a minimal medium to evaluate nutrient limitation on transposition. When required, media were supplemented with tetracycline (Tc) at a final concentration of 12.5 µg ml^−1^.

### DNA hybridization and sequence analyses

For hybridization probes, we amplified a complete copy of IS*801* or internal fragments of IS*Psy2*, IS*53* and IS*Psy24* from strain 1448A using specific primers (Table S2); these fragments were cloned in pGEM-T Easy and used as template for DNA amplification and labeling. Preparation of labeled probes with digoxigenin by PCR, Southern hybridization, and detection of hybridization signals were carried out with a DIG DNA labeling and detection kit (Roche Diagnostics) following the manufacturer's instructions.

To complete and update the genome annotation, the sequence of all the insertion sequences already found in *P. syringae* (IS Finder Database; http://www-is.biotoul.fr) [7], except IS*Psy27* whose sequence was not available, were individually compared to the genome of *P. syringae* pv. phaseolicola 1448A using the genomic blastn program [63] at the NCBI website. Likewise, the genome of strain 1448A was compared to the IS Finder Database using the blast programs to examine for the existence of new, not registered, insertion sequences. Following the criteria of the IS Finder repository, an insertion sequence was considered new when its deduced product was less than 98% similar and/or its DNA sequence less than 95% identical to any other previously identified insertion sequence in the databases. During annotation, an element was considered to be truncated when it lacked part or all of at least one of the terminal repeats, or when it contained internal deletions or insertions that affected the transposase promoter or caused premature stops or deletions larger than 100 nt in its reading frame.

Multalin [64] and Blast 2 Sequences [65] were used to search for homology among sequences and to produce sequence alignments. Annotation was done with Artemis [66] and genome comparisons with ACT [67] using WebACT [68]. All the transposable elements, and truncated fragments larger than 200 nt, were annotated in the genome of 1448A and numbered consecutively; truncated copies, or those having a premature stop codon or deletion in the transposase coding region, were numbered separately and identified with the letter T and a different color code (Annotation files S1, S2 and S3). Also, a list of revised annotations to the Pph 1448A genome can be found on the annotation updates page of the Pseudomonas-Plant Interaction web site (http://pseudomonas-syringae.org/) and in the IS-Finder genomes web site (http://www-genome.biotoul.fr/index.php). The sequence of MITE*Psy1* has been deposited in the EMBL databases under accession number FR714508.

### Trapping of insertion sequences

We originally used vector pGBG1, which allows entrapment of mobile elements when they insert into the *CI* repressor, thereby allowing the expression of resistance to tetracycline from the λ *pR* promoter [69]. However, the vector was inadequate for our purpose because it conferred constitutive resistance to tetracycline to *P. syringae* pv. phaseolicola. As an alternative strategy, therefore, we used vector pGEN500 to trap mobile elements by selection of sucrose resistance after inactivation of gene *sacB* [40].

Transformants of *P. syringae* pv. phaseolicola containing pGEN500 were selected on KMB plus tetracycline after electroporation, grown in the same conditions in liquid medium and stored at −80°C in 20% glycerol. To avoid the accumulation of insertions during routine transfer of cultures, the frequency of *sacB* inactivation was estimated, in general, using cultures started from cryopreserved transformants. To obtain pools of transformants, cells were incubated at 28°C for 2–4 h in KMB immediately after electroporation and a 100 µl aliquot was transferred to 5 ml of KMB+Tc. After overnight growth, aliquots of this culture were cryopreserved whereas other aliquots were directly used for the estimation of the transposition frequency. To isolate derivatives containing mobile elements inserted into *sacB*, cultures were diluted and spread onto SNA+Tc and the occurrence of insertions was monitored by examining changes in the mobility of pGEN500 in uncut plasmid preparations separated in 0.8% agarose gels [70]. The location and size of the corresponding insertions was analyzed by restriction digestion, PCR of the 5′ or the 3′ ends of the *sacB* gene and, in some cases, by DNA sequencing. PCR was carried out using primer pairs sacB5L1 (5′CCCGTAGTCTGCAAATCCTT3′) - sacB5R1 (5′GCCGTAATGTTTACCGGAGA3′) and sacB3L1 (5′GGTCAGGTTCAGCCACATTT3′) – sacB3R1 (5′GGCATTTTCTTTTGCGTTTT3′), designed from the published *sacB* sequence (accession no. X02730); these primer pairs allow for the amplification of the complete *sacB* CDS (1422 nt) plus its promoter in two overlapping fragments.

A working transposition frequency was estimated using the formula: [(number of sucrose and tetracycline resistant cfu per millilitre×0.92)/total number of tetracycline resistant cfu per millilitre]; in this formula, we multiplied by 0.92 because an average of 91.5% of the sucrose-resistant (suc^R^) clones among more than 500 analyzed (see Table 5, and data not shown) were found to originate by the insertion of a mobile element, whereas the remaining 8.5% was due to small deletions or individual nt changes in *sacB*. The frequency of transposition in rich and minimal media was estimated for five independent transformants of strain 1448A(pGEN500) grown overnight in KMB or MG, both supplemented with tetracycline; each experiment was repeated at least seven times. To estimate transposition from cells growing *in planta*, those five transformants were independently inoculated on leaves of bean (*Phaseolus vulgaris* L.) cv. Tendergreen, or fully expanded leaves of tobacco (*Nicotiana tabacum* cv. xanthi) as previously described [71], using plants maintained in growth chambers at 20°C, a photoperiod of 16/8 h day/night and 80% of humidity. Six hours after inoculation with bacterial suspensions adjusted to an OD_600_ of 0.5 (approximately 5×10^8^ cfu ml^−1^), excised leaf disks were ground in sterile 10 mM MgCl_2_ and appropriate dilutions plated on NA and SNA, both supplemented with tetracycline. The experiment was repeated at least three times. The analysis of transposition frequency data in different growing conditions was done using a two-way ANOVA test (p<0.05).

## Supporting Information

Figure S1 **Alignment of MITE** ***Psy1*** **with homologs in other bacteria.** Global alignments were done with Blast, and curated manually, between (upper sequence) a copy of MITE*Psy1* from *P. syringae* pv. phaseolicola 1448A (accession no. CP000058, positions 705671–705770) and (lower sequence) **A**) contig 32.3 (accession no. AEAL01000292.1) from the draft genome of *P. syringae* pv. actinidiae M302091; **B**) the genome of *P. syringae* pv. tomato DC3000 (accession no. AE016853), and **C**) the genome of *P. stutzeri* ATCC 17588 (accession no. CP002881; an identical alignment was obtained with the genome of the *Gammaproteobacterium* HdN1, accession no. FP929140, positions 593503–593601). **D**) Terminal inverted repeats of the MITE*Psy1* homolog present in *P. stutzeri* ATCC 17588 and the *Gammaproteobacterium* HdN1.
(DOC)

Figure S2 **Each transposition involves the movement of a single element.** Southern hybridization of DNA digested with PstI using a complete copy of IS*801* as a probe. Lanes contain genomic (Lane 1) or total plasmid DNA (Lane 7) from 1448A(pGEN500) or DNA isolated from clones containing independent insertions of mobile elements in gene *sacB* of pGEN500 as follows: two independent MITE*Psy1* insertions (Lanes 2 and 3); two independent insertions of IS*801* (Lanes 4 and 5), and IS*801* that recruited 1431 nt of adjacent DNA (Lane 6). Asterisks to the left of lanes indicate hybridization bands corresponding to the elements inserted in the vector. M, molecular weight marker (Kb Ladder, Agilent Technologies).
(PPT)

Table S1 Coding sequences annotated in the genome of *P. syringae* pv. phaseolicola (*Pph*) 1448A that are chimeras with or that are interrupted by mobile elements.
(DOC)

Table S2 List of primers used for the amplification of insertion sequences.
(DOC)

Annotation File S1 Annotation of insertion sequences, and fragments thereof larger than 200 nt, and MITEs in the chromosome of *P. syringae* pv. phaseolicola 1448A (accession no. CP000058). The file is in the format of a feature table (tab file) to be read as an entry with the Artemis browser.
(TAB)

Annotation File S2 Annotation of insertion sequences, and fragments thereof larger than 200 nt, and MITEs in plasmid p1448A-A of *P. syringae* pv. phaseolicola 1448A (accession no. CP000059). The file is in the format of a feature table (tab file) to be read as an entry with the Artemis browser.
(TAB)

Annotation File S3 Annotation of insertion sequences, and fragments thereof larger than 200 nt, in plasmid p1448A-B of *P. syringae* pv. phaseolicola 1448A (accession no. CP000060). The file is in the format of a feature table (tab file) to be read as an entry with the Artemis browser.
(TAB)

## References

[1] Siguier P, Filee J, Chandler M (2006). Insertion sequences in prokaryotic genomes.. Current Opinion in Microbiology.

[2] Mahillon J, Chandler M (1998). Insertion sequences.. Microbiology and Molecular Biology Reviews.

[3] Jackson RW, Vinatzer B, Arnold DL, Dorus S, Murillo J (2011). The influence of the accessory genome on bacterial pathogen evolution.. Mobile Genetic Elements.

[4] Craig NL, Craigie R, Gellert M, Lambowitz AM (2002). (2002) Mobile DNA II.

[5] McEvoy CRE, Falmer AA, van Pittius NCG, Victor TC, van Helden PD (2007). The role of IS*6110* in the evolution of *Mycobacterium tuberculosis*.. Tuberculosis.

[6] Sundin GW, Murillo J, Jackson RW (2009). Gene traders: characteristics of native plasmids from plant pathogenic bacteria.. Plant pathogenic bacteria: genomics and molecular biology.

[7] Siguier P, Perochon J, Lestrade L, Mahillon J, Chandler M (2006). ISfinder: the reference centre for bacterial insertion sequences.. Nucleic Acids Research.

[8] Bertels F, Rainey PB (2011). Within-genome evolution of REPINs: a new family of miniature mobile DNA in bacteria.. PLoS Genetics.

[9] Delihas N (2008). Small mobile sequences in bacteria display diverse structure/function motifs.. Molecular Microbiology.

[10] Mansfield JW (2009). From bacterial avirulence genes to effector functions via the *hrp* delivery system: an overview of 25 years of progress in our understanding of plant innate immunity.. Molecular Plant Pathology.

[11] Young JM (2010). Taxonomy of *Pseudomonas syringae*.. Journal of Plant Pathology.

[12] Sarkar SF, Guttman DS (2004). Evolution of the core genome of *Pseudomonas syringae*, a highly clonal, endemic plant pathogen.. Applied and Environmental Microbiology.

[13] Sarkar SF, Gordon JS, Martin GB, Guttman DS (2006). Comparative genomics of host-specific virulence in *Pseudomonas syringae*.. Genetics.

[14] Kim JF, Charkowski AO, Alfano JR, Collmer A, Beer SV (1998). Transposable elements and bacteriophage sequences flanking *Pseudomonas syringae* avirulence genes.. Molecular Plant-Microbe Interactions.

[15] Arnold DL, Jackson RW, Fillingham AJ, Goss SC, Taylor JD (2001). Highly conserved sequences flank avirulence genes: isolation of novel avirulence genes from *Pseudomonas syringae* pv. pisi.. Microbiology.

[16] Jackson RW, Athanassopoulos E, Tsiamis G, Mansfield JW, Sesma A (1999). Identification of a pathogenicity island, which contains genes for virulence and avirulence, on a large native plasmid in the bean pathogen *Pseudomonas syringae* pathovar phaseolicola.. Proceedings of the National Academy of Sciences USA.

[17] Joardar V, Lindeberg M, Jackson RW, Selengut J, Dodson R (2005). Whole-genome sequence analysis of *Pseudomonas syringae* pv. phaseolicola 1448A reveals divergence among pathovars in genes involved in virulence and transposition.. Journal of Bacteriology.

[18] Rodríguez-Palenzuela P, Matas I, Murillo J, López-Solanilla E, Bardaji L (2010). Annotation and overview of the *Pseudomonas savastanoi* pv. savastanoi NCPPB 3335 draft genome reveals the virulence gene complement of a tumour-inducing pathogen of woody hosts.. Environmental Microbiology.

[19] Buell CR, Joardar V, Lindeberg M, Selengut J, Paulsen IT (2003). The complete genome sequence of the *Arabidopsis* and tomato pathogen *Pseudomonas syringae* pv. *tomato* DC3000.. Proceedings of the National Academy of Sciences USA.

[20] Vivian A, Murillo J, Jackson RW (2001). The role of plasmids in phytopathogenic bacteria: mobile arsenals?. Microbiology.

[21] Feil H, Feil WS, Chain P, Larimer F, DiBartolo G (2005). Comparison of the complete genome sequences of *Pseudomonas syringae* pv. syringae B728a and pv. tomato DC3000.. Proceedings of the National Academy of Sciences USA.

[22] Sundin GW (2007). Genomic insights into the contribution of phytopathogenic bacterial plasmids to the evolutionary history of their hosts.. Annual Review of Phytopathology.

[23] Yamada T, Lee PD, Kosuge T (1986). Insertion sequence elements of *Pseudomonas savastanoi*: Nucleotide sequence and homology with *Agrobacterium tumefaciens* transfer DNA.. Proceedings of the National Academy of Sciences USA.

[24] Romantschuk M, Richter GY, Mukhopadhyay P, Mills D (1991). IS*801*, an insertion sequence element isolated from *Pseudomonas syringae* pathovar *phaseolicola*.. Molecular Microbiology.

[25] Kamiunten H, Inoue S, Yakabe Y, Iida S (2002). Characterization of IS*Psy2* and IS*Psy3*, newly identified insertion sequences in *Pseudomonas syringae* pv. eriobotryae.. Journal of General Plant Pathology.

[26] Landgraf A, Weingart H, Tsiamis G, Boch J (2006). Different versions of *Pseudomonas syringae* pv. *tomato* DC3000 exist due to the activity of an effector transposon.. Molecular Plant Pathology.

[27] Arnold DL, Lovell HC, Jackson RW, Mansfield JW (2011). *Pseudomonas syringae* pv. *phaseolicola*: from ‘has bean’ to supermodel.. Molecular Plant Pathology.

[28] Lovell HC, Jackson RW, Mansfield JW, Godfrey SAC, Hancock JT (2011). *In planta* conditions induce genomic changes in *Pseudomonas syringae* pv. phaseolicola.. Molecular Plant Pathology.

[29] Oguiza JA, Rico A, Rivas LA, Sutra L, Vivian A (2004). *Pseudomonas syringae* pv. phaseolicola can be separated into two genetic lineages distinguished by the possession of the phaseolotoxin biosynthetic cluster.. Microbiology.

[30] Führer ME, Navarro de la Fuente L, Rivas L, Hernández-Flores JL, Garcidueñas-Piña R, Fatmi M, Collmer A, Iacobellis NS, Mansfield JW, Murillo J (2008). Genetic relatedness among the different lineages of *Pseudomonas syringae* pv. phaseolicola.. *Pseudomonas syringae* pathovars and related pathogens Identification, epidemiology and genomics.

[31] Stevens C, Bennett MA, Athanassopoulos E, Tsiamis G, Taylor JD (1998). Sequence variations in alleles of the avirulence gene *avrPphE.R2* from *Pseudomonas syringae* pv. *phaseolicola* lead to loss of recognition of the AvrPphE protein within bean cells and a gain in cultivar-specific virulence.. Molecular Microbiology.

[32] Rivas LA, Mansfield J, Tsiamis G, Jackson RW, Murillo J (2005). Changes in race-specific virulence in *Pseudomonas syringae* pv. phaseolicola are associated with a chimeric transposable element and rare deletion events in a plasmid-borne pathogenicity island.. Applied and Environmental Microbiology.

[33] Szabo LJ, Mills D (1984). Integration and excision of pMC7105 in *Pseudomonas syringae* pv. *phaseolicola*: involvement of repetitive sequences.. Journal of Bacteriology.

[34] Garcillán-Barcia MP, de la Cruz F (2002). Distribution of IS*91* family insertion sequences in bacterial genomes: evolutionary implications.. FEMS Microbiology Ecology.

[35] Garcillán-Barcia MP, Bernales I, Mendiola MV, de la Cruz F, Craig NL, Craigie R, Gellert M, Lambowitz AM (2002). IS*91* rolling-circle transposition.. Mobile DNA II.

[36] Richter GY, Björklöf K, Romantschuk M, Mills D (1998). Insertion specificity and *trans*-activation of IS*801*.. Molecular and General Genetics.

[37] Bardaji L, Pérez-Martínez I, Rodríguez-Moreno L, Rodríguez-Palenzuela P, Sundin GW (2011). Sequence and role in virulence of the three plasmid complement of the model tumor-inducing bacterium *Pseudomonas savastanoi* pv. savastanoi NCPPB 3335.. PLoS ONE.

[38] Chen Y, Zhou F, Li G, Xu Y (2009). MUST: A system for identification of miniature inverted-repeat transposable elements and applications to *Anabaena variabilis* and *Haloquadratum walsbyi*.. Gene.

[39] Han Y, Wessler SR (2010). MITE-Hunter: a program for discovering miniature inverted-repeat transposable elements from genomic sequences.. Nucleic Acids Research.

[40] Ohtsubo Y, Genka H, Komatsu H, Nagata Y, Tsuda M (2005). High-temperature-induced transposition of insertion elements in *Burkholderia multivorans* ATCC 17616.. Applied and Environmental Microbiology.

[41] Gay P, Le Coq D, Steinmetz M, Berkelman T, Kado CI (1985). Positive selection procedure for the entrapment of insertion sequences elements in gram-negative bacteria.. Journal of Bacteriology.

[42] Nagy Z, Chandler M (2004). Regulation of transposition in bacteria.. Research in Microbiology.

[43] Drevinek P, Baldwin A, Lindenburg L, Joshi LT, Marchbank A (2010). Oxidative stress of *Burkholderia cenocepacia* induces insertion sequence-mediated genomic rearrangements that interfere with macrorestriction-based genotyping.. Journal of Clinical Microbiology.

[44] Valle J, Vergara-Irigaray M, Merino N, Penades JR, Lasa I (2007). σB regulates IS*256*-mediated *Staphylococcus aureus* biofilm phenotypic variation.. Journal of Bacteriology.

[45] Touchon M, Rocha EPC (2007). Causes of insertion sequences abundance in prokaryotic genomes.. Molecular Biology and Evolution.

[46] Lindeberg M, Myers CR, Collmer A, Schneider DJ (2008). Roadmap to new virulence determinants in *Pseudomonas syringae*: insights from comparative genomics and genome organization.. Molecular Plant-Microbe Interactions.

[47] Wallden K, Rivera-Calzada A, Waksman G (2010). Type IV secretion systems: versatility and diversity in function.. Cellular Microbiology.

[48] Norman A, Hansen LH, Sørensen SJ (2009). Conjugative plasmids: vessels of the communal gene pool.. Philosophical Transactions of the Royal Society B: Biological Sciences.

[49] Mendiola MV, Bernales I, de la Cruz F (1994). Differential roles of the transposon termini in IS*91* transposition.. Proceedings of the National Academy of Sciences USA.

[50] Toleman MA, Bennett PM, Walsh TR (2006). ISCR elements: novel gene-capturing systems of the 21st century?. Microbiology and Molecular Biology Reviews.

[51] Stavrinides J, Ma W, Guttman DS (2006). Terminal reassortment drives the quantum evolution of type III effectors in bacterial pathogens.. PLoS Pathogens.

[52] Yan S, Liu H, Mohr TJ, Jenrette J, Chiodini R (2008). Role of recombination in the evolution of the model plant pathogen *Pseudomonas syringae* pv. tomato DC3000, a very atypical tomato strain.. Applied and Environmental Microbiology.

[53] Burt A, Trivers R (2006). Genes in conflict: The biology of selfish genetic elements.

[54] Zhou F, Tran T, Xu Y (2008). Nezha, a novel active miniature inverted-repeat transposable element in cyanobacteria.. Biochemical and Biophysical Research Communications.

[55] Robertson AE, Wechter WP, Denny TP, Fortnum BA, Kluepfel DA (2004). Relationship between avirulence gene (*avrA*) diversity in *Ralstonia solanacearum* and bacterial wilt incidence.. Molecular Plant-Microbe Interactions.

[56] Poirel L, Carrer A, Pitout JD, Nordmann P (2009). Integron mobilization unit as a source of mobility of antibiotic resistance genes.. Antimicrobial Agents and Chemotherapy.

[57] Stavrinides J, Guttman D (2004). Nucleotide sequence and evolution of the five-plasmid complement of the phytopathogen *Pseudomonas syringae* pv. maculicola ES4326.. Journal of Bacteriology.

[58] Sambrook J, Fritsch EF, Maniatis T (1989). Molecular Cloning: a Laboratory Manual.

[59] Teverson DM (1991). Genetics of pathogenicity and resistance in the halo-blight disease of beans in Africa [Ph. D. Thesis].

[60] Taylor JD, Teverson DM, Allen DJ, Pastor-Corrales MA (1996). Identification and origin of races of *Pseudomonas syringae* pv. *phaseolicola* from Africa and other bean growing areas.. Plant Pathology.

[61] King EO, Ward NK, Raney DE (1954). Two simple media for the demonstration of pyocyanin and fluorescein.. Journal of Laboratory and Clinical Medicine.

[62] Keane PJ, Kerr A, New. PB (1970). Crown gall of stone fruit. II. Identification and nomenclature of *Agrobacterium* isolates.. Australian Journal of Biological Sciences.

[63] Cummings L, Riley L, Black L, Souvorov A, Resenchuk S (2002). Genomic BLAST: custom-defined virtual databases for complete and unfinished genomes.. FEMS Microbiology Letters.

[64] Corpet F (1988). Multiple sequence alignment with hierarchical clustering.. Nucleic Acids Research.

[65] Tatusova TA, Madden TL (1999). BLAST 2 SEQUENCES, a new tool for comparing protein and nucleotide sequences.. FEMS Microbiology Letters.

[66] Rutherford K, Parkhill J, Crook J, Horsnell T, Rice P (2000). Artemis: sequence visualisation and annotation.. Bioinformatics.

[67] Carver TJ, Rutherford KM, Berriman M, Rajandream M-A, Barrell BG (2005). ACT: the Artemis Comparison Tool.. Bioinformatics.

[68] Abbott JC, Aanensen DM, Rutherford K, Butcher S, Spratt BG (2005). WebACT-an online companion for the Artemis Comparison Tool.. Bioinformatics.

[69] Miché L, Fauré D, Blot M, Cabanne-Giuli E, Balandreau J (2001). Detection and activity of insertion sequences in environmental strains of *Burkholderia*.. Environmental Microbiology.

[70] Sesma A, Sundin GW, Murillo J (1998). Closely related replicons coexisting in the phytopathogen *Pseudomonas syringae* show a mosaic organization of the replication region and altered incompatibility behavior.. Applied and Environmental Microbiology.

[71] Harper S, Zewdie N, Brown IR, Mansfield JW (1987). Histological, physiological and genetical studies of the responses of leaves and pods of *Phaseolus vulgaris* to three races of *Pseudomonas syringae* pv. *phaseolicola* and to *Pseudomonas syringae* pv. *coronafaciens*.. Physiological and Molecular Plant Pathology.

