# Supplementary material for: Miniature Transposable Sequences Are Frequently Mobilized in the Bacterial Plant Pathogen Pseudomonas syringae pv. phaseolicola
Source: PLoS One. 2011 Oct 10;6(10):e25773. doi: 10.1371/journal.pone.0025773 (PMC3189936; doi:10.1371/journal.pone.0025773)
Supplement: Figure S1 — Alignment of MITE Psy1 with homologs in other bacteria. Global alignments were done with Blast, and curated manually, between (upper sequence) a copy of MITEPsy1 from P. syringae pv. phaseolicola 1448A (accession no. CP000058, positions 705671–705770) and (lower sequence) A) contig 32.3 (accession no. AEAL01000292.1) from the draft genome of P. syringae pv. actinidiae M302091; B) the genome of P. syringae pv. tomato DC3000 (accession no. AE016853), and C) the genome of P. stutzeri ATCC 17588 (accession no. CP002881; an identical alignment was obtained with the genome of the Gammaproteobacterium HdN1, accession no. FP929140, positions 593503–593601). D) Terminal inverted repeats of the MITEPsy1 homolog present in P. stutzeri ATCC 17588 and the Gammaproteobacterium HdN1. (DOC) [file pone.0025773.s001.doc]

**A)**

MITE*Psy1* 8 CTGAAAAAGCCTTTTCTTCAAAAGTCGAAGCCAGTAAATATAGGCGCTCCAGCCCGGTTC 67

|||||||||||| ||| ||||| |||||||||||||||| | | |||| |||| |

M302091 16712 CTGAAAAAGCCTCTTCGTCAAACATCGAAGCCAGTAAATACACGTGCTC---TCCGGCAC 16656

MITE*Psy1* 68 CTCTCCAAAAAAATGGGCTTTTTCAGAGGAT 98

||| | |||| | || | ||||||||| ||

M302091 16655 GTCTGC-AAAAGAGGGACGTTTTCAGAGAAT 16626

**B)**

MITE*Psy1* 45 ATATAGGCGCTCCAGCCCGGTTCCTCTCCAAAAAAATGGGCTTTTTCAGAGG 96

||| |||||||||||||||| || ||| ||||||||||||||||||||||

DC3000 6147431 ATAC-GGCGCTCCAGCCCGGTCTCTTTCC-AAAAAATGGGCTTTTTCAGAGG 6147480

**C)**

MITE*Psy1* 1 GGAAGGTCTGAAAAAGCCTTTTCTTCAAAAGTCGAAGCCAGTAAATATAGGCGCTCCAG- 59

||| |||||||||||| | ||||| |||| | || ||||||| || | | | |||

Pstu 134024 GGATGGTCTGAAAAAGTCATTTCTCGAAAAATGGAGCCCAGTAACCATGCGGGATACAGG 133965

MITE*Psy1* 60 --CCCGGTTCCTCTCCAAAAAAATGGGCTTTTTCAGAGGATAC 100

||||| || ||||| |||||||||||||| | |

Pstu 133964 GCAGCGGTT----TCGCGAAAAACGGGCTTTTTCAGAGCTTCC 133936

**D)**

1 GGATGGTCTGAAAAAGTC 18

||| | |||||||||| |

99 GGAAGCTCTGAAAAAGCC 82
