# Supplementary figures and images for: Miniature Transposable Sequences Are Frequently Mobilized in the Bacterial Plant Pathogen Pseudomonas syringae pv. phaseolicola
Source: PLoS One. 2011 Oct 10;6(10):e25773. doi: 10.1371/journal.pone.0025773 (PMC3189936; doi:10.1371/journal.pone.0025773)

## Slide 1
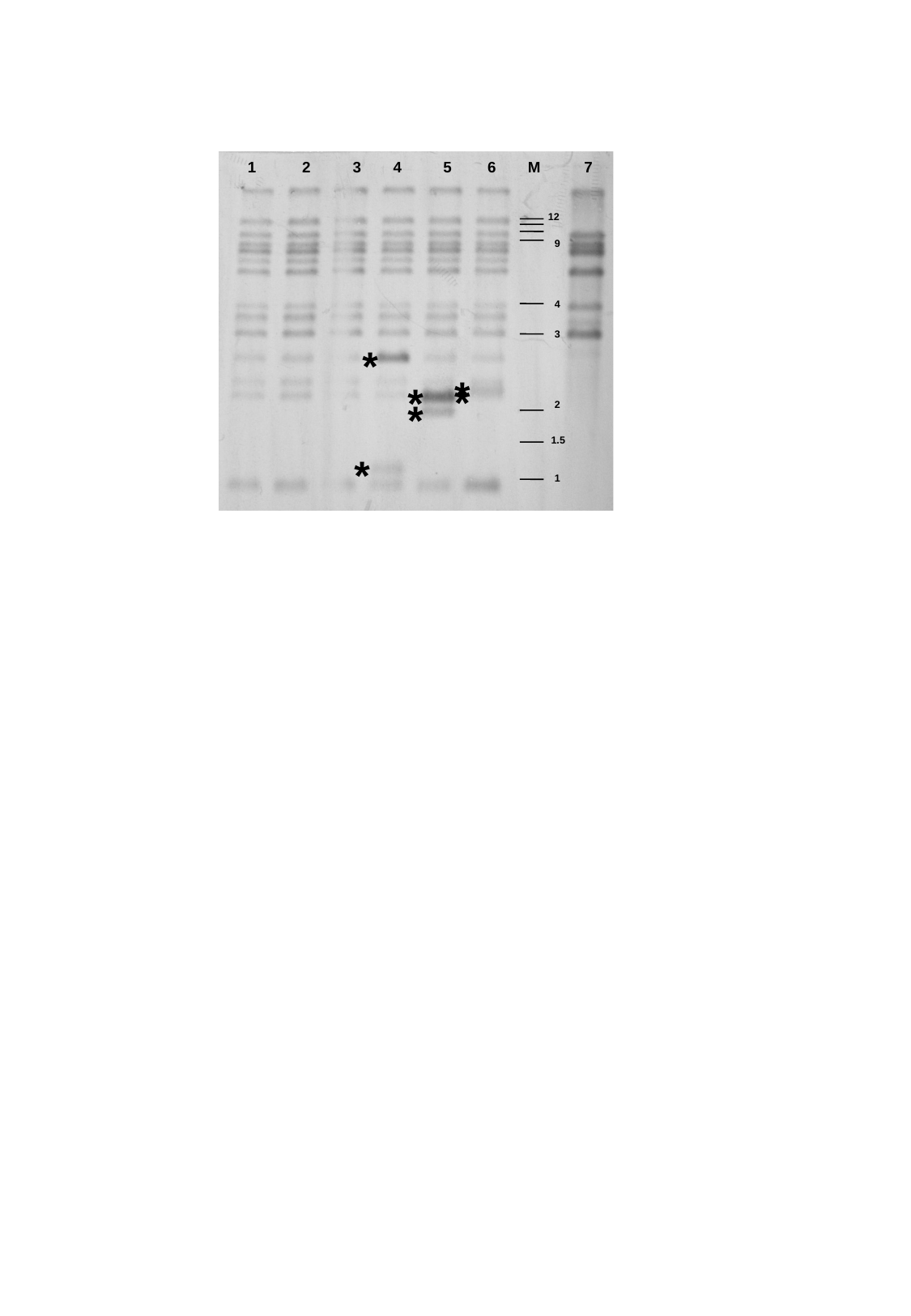

1
2
3
4
5
6
M
7
12
9
4
3
*
*
*
*
*
2
1.5
*
1

Supplement: Figure S2 — Each transposition involves the movement of a single element. Southern hybridization of DNA digested with PstI using a complete copy of IS801 as a probe. Lanes contain genomic (Lane 1) or total plasmid DNA (Lane 7) from 1448A(pGEN500) or DNA isolated from clones containing independent insertions of mobile elements in gene sacB of pGEN500 as follows: two independent MITEPsy1 insertions (Lanes 2 and 3); two independent insertions of IS801 (Lanes 4 and 5), and IS801 that recruited 1431 nt of adjacent DNA (Lane 6). Asterisks to the left of lanes indicate hybridization bands corresponding to the elements inserted in the vector. M, molecular weight marker (Kb Ladder, Agilent Technologies). (PPT) [file pone.0025773.s002.ppt]
