# Supplementary material for: Miniature Transposable Sequences Are Frequently Mobilized in the Bacterial Plant Pathogen Pseudomonas syringae pv. phaseolicola
Source: PLoS One. 2011 Oct 10;6(10):e25773. doi: 10.1371/journal.pone.0025773 (PMC3189936; doi:10.1371/journal.pone.0025773)
Supplement: Table S1 — Coding sequences annotated in the genome of P. syringae pv. phaseolicola (Pph) 1448A that are chimeras with or that are interrupted by mobile elements. (DOC) [file pone.0025773.s003.doc]

**Table S1.** Coding sequences annotated in the genome of *P. syringae* pv. phaseolicola (*Pph*) 1448A that are chimeras with or that are interrupted by mobile elements.^a^

| Locus | Closest homolog (accession no.) | Size (aa) | | Product | Number of aa from mobile element^b^ | | Mobile element |
| --- | --- | --- | --- | --- | --- | --- | --- |
|  |  | in *Pph* | in homolog |  | start | end |  |
| **Chimeras** | |  |  |  |  |  |  |
| PSPPH_0008^c^ | EFW82723 | 452 | 787 | oxidoreductase, alpha (molybdopterin) subunit, fusion |  | 16 (351) | IS*801* |
| PSPPH_0015^d^ | EFW86903 | 205 | 224 | hypothetical protein |  | 15 (34) | IS*801* |
| PSPPH_0017 | ZP_05639629.1 | 542 | 787 | oxidoreductase, alpha (molybdopterin) subunit, fusion |  | 16 (261) | IS*801* |
| PSPPH_0115 | EFW87038 | 167 | 343 | lipoprotein, putative |  | 3 (179) | IS*Psy2* |
| **PSPPH_0340** | ZP_05635939.1 | 321 | 247 | GlnQ1; amino acid ABC transporter, ATP-binding protein |  | 79 (5) | IS*Psy17* |
| PSPPH_0605 | ZP_05636412.1 | 231 | 294 | O-antigen ABC transporter, permease protein, putative |  | 2 (98) | IS*Psy19* |
| **PSPPH_0618** | EFW88007 | 227 | 290 | sensory box/GGDEF domain/EAL domain protein |  | 79 (142) | IS*Psy17* |
| PSPPH_0823 | ZP_07265874.1 | 482 | 419 | membrane protein, putative |  | 80 (27) | IS*Psy17* |
| PSPPH_0898 | ZP_07003659.1 | 122 | 144 | conserved hypothetical protein |  | 14 (36) | IS*Psy19* |
| PSPPH_1063 | ZP_07003819.1 | 205 | 198 | membrane protein, putative |  | 14 (7) | IS*Psy1* *7* |
| PSPPH_1139 | ZP_06461567.1 | 210 | 310 | conserved hypothetical protein |  | 3 (103) | IS*53* |
| PSPPH_1142 | EFW81912 | 401 | 415 | hypothetical protein |  | 29 (43) | IS53 |
| PSPPH_1445 | No homologs found | 41 | - | hypothetical protein | 26 |  | IS*Psy22* |
| PSPPH_1489 | ZP_05640026.1 | 323 | 358 | GGDEF domain protein | 23 (58) |  | IS*Psy19* |
| PSPPH_1504 | YP_236820.1 | 69 | 64 | global regulator (carbon storage regulator) |  | 11 (26) | IS*Psy17* |
| PSPPH_1524 | ABR13392.1 | 209 | 385 | site-specific recombinase, phage integrase family, putative |  | 3 (179) | IS*Psy16* |
| PSPPH_1525^e^ | Many | 2571 | variable | hypothetical protein |  | 6 | IS*Psy16* |
| PSPPH_1530 | EFI00414.1 | 144 | 532 | polygalacturonase |  | 3 (392) | IS*53* |
| PSPPH_1843 | ZP_06480584.1 | 505 | 535 | conserved hypothetical protein |  | 31 (61) | IS*Psy24* |
| PSPPH_1901 | ZP_05638425.1 | 507 | 492 | membrane protein, putative | 10 (0) |  | IS*Psy17* |
| PSPPH_2285 | YP_262472.1 | 55 | 37 | hypothetical protein | The whole CDS is within IS sequence | | IS*Psy25* |
| PSPPH_2440 | ZP_06460001.1 | 121 | 438 | DNA replication and repair protein RecF |  | 12 (329) | IS*Psy17* |
| PSPPH_2731 | ZP_06494991.1 | 123 | 198 | conserved domain protein |  | 14 (90) | IS*Psy19* |
| PSPPH_2800 | YP_001749219.1 | 78 | 91 | transcriptional regulator, PbsX family |  | 1 (16) | IS*Psy24* |
| PSPPH_2922 | ZP_05640056.1 | 207 | 506 | peptide ABC transporter, periplasmic peptide-binding protein |  | 7 (303) | IS*801* |
| PSPPH_2924 | EGH90444 | 211 | 281 | hypothetical protein | 15 (85) |  | IS*801* |
| PSPPH_2980 | ZP_07266387.1 | 318 | 391 | two-component system sensor protein | 12 (86) |  | IS*Psy17* |
| PSPPH_3030 | EFW84348 | 256 | 279 | putative lipoprotein |  |  | IS*801* |
| PSPPH_3578 | ZP_05636784.1 | 264 | 194 | conserved hypothetical protein |  | 79 (15) | IS*Psy17* |
| PSPPH_3580^f^ | ZP_05636783.1 | 217 | 308 | conserved hypothetical protein | 10 (-) | 3 (123) | IS*Psy17* and IS*Psy2* |
| PSPPH_3641 | ZP_07006078.1 | 276 | 376 | hypothetical protein | 10 (110) |  | IS*Psy17* |
| PSPPH_3976 | ZP_07006683.1 | 597 | 595 | amidase family protein |  | 12 (10) | IS*Psy17* |
| PSPPH_4158 | ZP_05637539.1 | 66 | 155 | conserved hypothetical protein | 5 (71) | 12 (33) | IS*Psy19* and IS*Psy17* |
| PSPPH_4269 | ZP_05636160.1 | 379 | 414 | unnamed protein product |  | 12 (47) | IS*Psy17* |
| PSPPH_4274 | ZP_05636163.1 | 326 | 369 | F-box domain protein | 23 (94) |  | IS*Psy19* |
| PSPPH_4286 | ZP_06461287.1 | 32 | 162 | conserved hypothetical protein |  | 7 (137) | IS*Psy19* |
| PSPPH_4560 | EGH68474 | 1848 | 2944 | filamentous hemagglutinin |  | 13 (1048) | IS*Psy4* |
| PSPPH_4991 | ZP_05639789.1 | 159 | 207 | glycosyl hydrolase, family 25 |  | 5 (53) | IS*Psy2* |
| PSPPH_A0017 | PSPSV_C0046  (FR820587) | 138 | 175 | hypothetical protein | 37 (75) |  | MITE*Psy1* |
| PSPPH_A0059 | ZP_06482943 | 75 | 189 | hypothetical protein |  | 1 (72) | IS*Psy2* |
| PSPPH_A0133^e^ | Many | 2151 | variable | hypothetical protein |  | 6 | IS*Psy16* |
| PSPPH_A0144 | YP_272282 | 123 | 162 | conjugal transfer protein | 1 (39) |  | IS*Psy26* |
| **Interrupted** | |  |  |  |  |  |  |
| **PSPPH_0045** | ZP_06458562 | 97 | 222 | hypothetical protein | 0 (125) |  | IS*Psy17* |
| **PSPPH_0046** |  | 149 |  |  |  | 26 (99) |  |
| PSPPH_0770 | YP_001778265.1 | 762 | 1194 | response regulator/sensor histidine kinase, putative | 39 (511) |  | IS*Psy19 and* MITE*Psy1* |
| PSPPH_0777 |  | 577 |  |  |  | 32 (781) |  |
| PSPPH_0893^g^ | ZP_05635966.1 | 62 | 673 | acyltransferase family protein | 6 |  | IS*801* |
| PSPPH_0895^g^ |  | 206 |  |  |  | 22 |  |
| **PSPPH_1596** | Many | 122 | variable | Ice nucleation protein |  |  | IS*Psy17* |
| **PSPPH_1598** |  | 1138 |  |  |  |  |  |
| **PSPPH_1991** | EGH14971 | 426 | 636 | response regulator receiver:ATP-binding region | 0 (200) |  | IS*Psy17* |
| **PSPPH_1993** |  | 195 |  |  |  | 10 (451) |  |
| PSPPH_2005 | ZP_06478048.1 | 302 | 683 | conserved hypothetical protein |  | 3 (382) | IS*Psy19* |
| PSPPH_2007 |  | 408 |  |  | 25 (301) |  |  |
| PSPPH_2311 | ZP_05638631.1 | 1157 | 2608 | insecticidal toxin complex protein, putative | 12 (1462) |  | IS*Psy17* |
| PSPPH_2313 |  | 288 |  |  |  | 0 (2320) |  |
| **PSPPH_2169** | EFW80728 | 319 | 298 | regulatory protein, LysR:LysR |  | 80 (59) | IS*Psy17* |
| **PSPPH_2171** |  | 71 |  |  | 10 (227) |  |  |
| **PSPPH_3440** | YP_233307 | 434 | 1126 | hypothetical protein |  | 12 (705) | IS*Psy17* |
| **PSPPH_3442** |  | 273 |  |  | 35 (889) |  |  |
| **PSPPH_4835** | EGH92078 | 236 | 441 | outer membrane porin | 12 (217) |  | IS*Psy17* |
| **PSPPH_4837** |  | 227 |  |  |  | 11 (224) |  |
| PSPPH_A0045 | YP_571076 | 117 | 396 | hypothetical protein | 6 |  | IS*801* |
| PSPPH_A0047 |  | 274 |  |  |  | 16 |  |

^a^ Lines shaded in gray correspond to chimeric loci that are likely pseudogenes, because they are interrupted in the 5’ end. Loci in bold and underlined were reported previously (Joardar et al. 2005, J. Bacteriol. 187:6488).

^b^ Number of amino acids that correspond to the mobile element in the CDS of *P. syringae* pv. phaseolicola 1448A, in brackets is the number of extra amino acids that are present in the closest homolog and that were probably lost due to the truncation.

^c^ The 5’ end of the PSPPH_0008 CDS is missing at least 153 nt in comparison with its closest homolog.

^d^ The C terminal end of the deduced product of locus PSPPH_0015 differs from homologs after position 159, suggesting that sequence has suffered reorganizations.

^e^ Homologs of loci PSPPH_1525 and PSPPH_A0133 show very variable lengths, usually because of variations in the number of internal repeats; therefore, we cannot determine what would have been the wild type length of these loci before the insertions of IS*Psy16*.

^f^ The annotation of PSPPH_3580 differs from homologs in other *P. syringae* in having 90 extra nt in the 5' end, which overlap IS*Psy17*.

^g^ Loci PSPPH_0893, PSPPH_0895 and PSPPH_0896 were probably a single continuous CDS, homologous to PsyrptA_020100001573, which has been disrupted by the insertion of IS*801* and other events.
