# Supplementary material for: Miniature Transposable Sequences Are Frequently Mobilized in the Bacterial Plant Pathogen Pseudomonas syringae pv. phaseolicola
Source: PLoS One. 2011 Oct 10;6(10):e25773. doi: 10.1371/journal.pone.0025773 (PMC3189936; doi:10.1371/journal.pone.0025773)
Supplement: Table S2 — List of primers used for the amplification of insertion sequences. (DOC) [file pone.0025773.s004.doc]

**Table S2.** List of primers used for the amplification of insertion sequences.

|  | | | | |
| --- | --- | --- | --- | --- |
| IS (size, nt) | Primer | Sequence (5’-3’)Bardaji et al | Position^a^ | Product size, nt |
| IS*801* (1512) | IS801c-F | CGTCCCCTCCGAACTCAT | 19521 | 1555 |
|  | IS801.11155_nR | ACGCGACCTGCAGAACAG | 21076 |  |
| IS*53* (2572) | IS53-F | GCAGGAGAACACAGGGTGAT | 92 | 2323 |
|  | IS53-R | GGATCAAAATGGCTCATCGT | 2414 |  |
| IS*Psy24* (1235) | ISPsy24-F | GACACCCATTTAGGCGAGAA | 22 | 1058 |
|  | ISPsy24-R | CGGTCGGTATCCACTCAGTT | 1079 |  |
| IS*Psy2* (1194) | ISPsy2-R2 | GGAAACTCTGAAAAAGACTTCCT | 1 | 1066 |
|  | ISPsy2-F | CGGTAACCATTTCCTGCATT | 1066 |  |

^a^ Numbers indicate the position of the first nt of each primer within the insertion sequence, except for the primers used to amplify IS*801*, which are located outside the element; in this case, numbers correspond to their position within the genome of Pph 1448A (accession no. CP000058).
